# Endoplasmic Reticulum Stress Is Involved in the Response of Human Laryngeal Carcinoma Cells to Carboplatin but Is Absent in Carboplatin-Resistant Cells

**DOI:** 10.1371/journal.pone.0076397

**Published:** 2013-09-23

**Authors:** Anamaria Brozovic, Lidija Vuković, Darija Stupin Polančac, Istvan Arany, Beate Köberle, Gerhard Fritz, Željka Fiket, Dragomira Majhen, Andreja Ambriović-Ristov, Maja Osmak

**Affiliations:** 1 Division Of Molecular Biology, Ruđer Bošković Institute, Zagreb, Croatia; 2 Galapagos Research Center Ltd., Zagreb, Croatia; 3 Department Of Pediatrics, University Of Mississippi Medical Center, Jackson, Massachusetts, United States of America; 4 Institute For Toxicology, University Medical Centre Of The Johannes Gutenberg University Mainz, Mainz, Germany; 5 Institute For Toxicology, Heinrich Heine University Düsseldorf, Düsseldorf, Germany; 6 Division For Marine And Environmental Research, Ruđer Bošković Institute, Zagreb, Croatia; Complutense University, Spain

## Abstract

The major obstacle of successful tumor treatment with carboplatin (CBP) is the development of drug resistance. In the present study, we found that following treatment with CBP the amount of platinum which enters the human laryngeal carcinoma (HEp2)-derived CBP-resistant (7T) cells is reduced relative to the parental HEp2. As a consequence, the formation of reactive oxidative species (ROS) is reduced, the induction of endoplasmic reticulum (ER) stress is diminished, the amount of inter- and intrastrand cross-links is lower, and the induction of apoptosis is depressed. In HEp2 cells, ROS scavenger tempol, inhibitor of ER stress salubrinal, as well as gene silencing of ER stress marker CCAAT/enhancer-binding protein (CHOP) increases their survival and renders them as resistant to CBP as 7T cell subline but did not influence the survival of 7T cells. Our results suggest that in HEp2 cells CBP-induced ROS is a stimulus for ER stress. To the contrary, despite the ability of CBP to induce formation of ROS and activate ER stress in 7T cells, the cell death mechanism in 7T cells is independent of ROS induction and activation of ER stress. The novel signaling pathway of CBP-driven toxicity that was found in the HEp2 cell line, i.e. increased ROS formation and induction of ER stress, may be predictive for therapeutic response of epithelial cancer cells to CBP-based therapy.

## Introduction

Carboplatin (*cis*-Diammine(1,1-cyclobutanedicarboxylato)platinum(II); CBP) is an important drug used to treat different types of epithelial tumors [1], [2], [3]. Although, CBP is effective in many cancers, its long-term clinical usage has been limited because of the development of tumor drug resistance [4], [5], [6]. Establishment of epithelial tumor cell lines resistant to clinically relevant doses of CBP is a useful approach in the identification of potential molecular targets in order to overcome drug resistance, and these biomarkers may be useful in predicting CBP sensitivity or resistance. Several molecular mechanisms have been described to be involved in CBP resistance: (a) diminished drug accumulation, (b) elevated drug inactivation, (c) DNA repair or elevated DNA damage tolerance, (d) enhanced expression of anti-apoptotic genes, and (e) inactivation of the p53 pathway (all reviewed in [5], [7]). However, none of these molecular mechanisms was found to be the single dominant factor determining CBP resistance. More likely, CBP resistance is a result of multiple mechanisms activated in parallel upon drug treatment.

Although only a small percentage of cell-associated CBP is found in the DNA fraction [8], it is generally accepted that DNA damage is a main cause of platinum-induced cell death [5], [9], [10], [11], [12]. However, there is evidence showing that CBP can also induce oxidative stress [13]. Carboplatin causes reactive oxidative species (ROS) formation, depletes the cellular pool of antioxidants, and causes oxidative injury in the cochleae of rats [14], specifically in the interior collicolus, leading to hearing loss [15]. The overexpression and activation of the heme oxygenase (HO)-1, which is an inducible form of HO in response to oxidative stress, provided protection against CBP-induced injury [16].

Reactive oxidative species can induce the endoplasmic reticulum (ER) stress response [17], a consequence of the accumulation of unfolded and/or misfolded proteins in the ER lumen. Different types of cellular stresses such as nutrient deprivation, disturbance of calcium flux or alterations in the glycosylation status can cause ER stress. ER stress further triggers signaling pathways which promote cell death [18]. Cisplatin (cDDP), the parent compound of CBP, has been shown to cause ER stress and DNA damage-independent cell death [19], [20]. Further, in renal tubular epithelial cells caspase-12, a marker of ER stress, plays a pivotal role in cDDP-induced cell death [21].

We hypothesized that CBP-induced ROS is one of the triggers of ER stress. To support this hypothesis, ROS formation and the induction of ER stress were comparatively investigated in human laryngeal carcinoma parental HEp2 cells and their CBP-resistant 7T subline following treatment with CBP. Additionally, we examined the influence of ROS scavenger tempol, specific inhibition of ER stress by salubrinal and RNAi mediated downregulation of one of the marker of ER stress on cellular sensitivity to CBP.

## Materials and Methods

### Cell lines

Human laryngeal carcinoma (HEp2) cells were obtained from a cell culture bank (GIBCO/BRL Life Technologies, Germany; ATCC Nr. CCL-23) and their authenticity was confirmed by Leibniz-Institut DSMZ-Deutsche Sammlung von Mikroorganismen und Zellkulturen GmbH. A human laryngeal carcinoma cell subline resistant to carboplatin (7T) was selected by CBP (Sigma-Aldrich, Germany; Cat. Nr. C2538) treatment of parental HEp2 cells [22]. Briefly, the cells were treated continuously with CBP for five consecutive days, and cultured in drug-free medium until surviving cells recovered and achieved a normal growth rate. Thereafter, they were treated again for five days with increased concentrations of CBP. These five-day cycles were repeated until the clinical relevant concentration of 9 mg/L (24 µM) of CBP was reached. The 7T subline was then isolated from the CBP-resistant cell population [22]. Both cell lines were grown in Dulbecco's medium (Gibco BRL Life Technologies; Cat. Nr. 11965) supplemented with 10% fetal bovine serum (Sigma-Aldrich; Cat. Nr. F7524) and cultured in a humidified atmosphere of 5% CO_2_ at 37°C.

### Determination of cell survival

The sensitivity of HEp2 and 7T cells upon CBP treatment was determined using the MTT colorimetric assay [23] (Sigma-Aldrich; Cat. Nr. M2128). Briefly, cells were seeded in 96-well tissue culture plate (2.5×10^3^ cells/well) and allowed to attach overnight, followed by, if indicated, two hours pretreatment with tempol or salubrinal (Santa Cruz Biotechnology; Cat. Nr. sc-200825, Cat. Nr. sc-202332) and/or only treatment with different concentrations of CBP. Following 72 h drug incubation, the MTT reagent was added, the resulting MTT-formazan product was dissolved in DMSO and absorbance measured using a microplate reader (Awareness Technology Inc., USA) at 545 nm.

### Determination of apoptosis

Apoptosis was determined using fluorescence microscopy. Untreated and treated cells were trypsinized, harvested, and collected by centrifugation. The cells were resuspended in a Dulbecco's medium at a cell density of 1×10^6^ cells/mL and stained by mixing 10 µL of a cell suspension with 4 µL of 1% propidium iodide (Sigma-Aldrich; Cat. Nr. P4170) and 2 µL of 1% ethidium bromide (Sigma-Aldrich; Cat. Nr. E8751). Apoptotic cells with characteristic morphology were identified under a fluorescence microscope. At least 300 cells were counted per sample. The percentage of apoptotic cells was calculated relative to the number of viable and necrotic cells.

### Preparation of total cell extracts

The cells were trypsinized and harvested by centrifugation, washed with PBS and resuspended in sonication buffer (20 mM Tris/HCl, pH 8.5, 1 mM EDTA, 5% glycerin, 1 mM DTT, 0.5 mM PMSF). After sonication, cell debris was removed by centrifugation (15 min, 20,000× *g* at 4°C). The supernatants containing total cellular proteins were collected and protein concentration was determined.

### Western blot analysis

30 µg of total cellular proteins were loaded onto a 10% SDS polyacrylamide gel and run for 2 h at 35 mA. Separated proteins were transferred onto a 0.2 µm nitrocellulose membrane (Schleicher and Schüll, Germany; Cat. Nr. NBA083C001EA) in a Bio-Rad blot cell (Bio-Rad, USA) using buffer consisting of 25 mM Tris/HCl, 86 mM glycine and 20% methanol. To avoid nonspecific binding, the membrane was incubated in blocking buffer (5% nonfat dry milk, 0.1% Tween 20 in PBS) for one hour at room temperature. Incubation with monoclonal and phosphor-polyclonal antibodies was performed overnight at 4°C. The incubation with polyclonal antibodies was performed for two hours at room temperature. Following primary antibodies were used: activating transcription factor 4 (ATF4), eukaryotic initiation factor 2 alpha (eIF2α), X-box binding protein 1 (XBP-1) (Santa Cruz Biotechnology; Cat. Nr. sc-200, Cat. Nr. sc-30882, Cat. Nr. sc-7160), p-histone H2AX (EMD Millipore, USA; Cat. Nr. 05-636), CCAAT/-enhancer-binding protein homologous protein (CHOP), glucose-regulated protein (Grp78), phospho-eIF2α (p-eIF2α; Cell Signaling Technology, USA; Cat. Nr. 2895, Cat. Nr. 3177, Cat. Nr. 9721). After washing with 0.01% Tween-20 in PBS and incubation with corresponding horseradish-peroxidase-coupled secondary antibody (Amersham Pharmacia Biotech, Germany; Cat. Nr. NA931 and Cat. Nr. NA934 and Santa Cruz Biotechnology; Cat. Nr. sc-2020), proteins were visualized with ECL (Amersham Pharmacia Biotech; Cat. Nr. RPN2106) according to the manufacturer's protocol. All membranes were incubated with anti-extracellular-signal-regulated kinases 1/2 or 2 (anti-ERK1/2 or anti-ERK2) (Santa Cruz Biotechnology, USA; Cat. Nr. sc-153, Cat. Nr. sc-292838) antibody to confirm equal protein loading. ERK2 or ERK1/2 were used as loading controls since no changes in total ERK1 and ERK2 expression was detected upon exposure of cells to different drugs [24], [25].

### South-western slot-blot analysis

Genomic DNA was isolated from sub-confluent cells by use of the QIA(amp) blood mini kit (Qiagen, Germany). 1 µg DNA was transferred to a positively charged nylon membrane (Hybond plus, Amersham Pharmacia Biotech; Cat. Nr. RPN203B) by vacuum slot-blotting, denatured with 0.3 M NaOH, neutralized with 5× SSC, and fixed by baking the membrane for 2 h at 80°C. Equal DNA loading was ensured by measurement of DNA concentration and by densitometrical determination of DNA concentration on agarose gel prior to spotting. Furthermore, equal DNA loading on the nylon membrane upon chemiluminescence was confirmed by staining the membrane in an aqueous solution of 0.5 µg/mL ethidium bromide for about 30 min. Upon staining, the membrane was rinsed in water for 15 min and the integrated ethidium bromide was visualized by a transilluminator, photographed and the spots compared by densitometry. The antibody specifically detecting 1,2-GG intrastrand cross-links induced by CBP was kindly provided by J. Thomale (Essen, Germany) and was described elsewhere [26]. The western blot procedure was performed as described above.

### Isolation of RNA, semi-quantitative and real-time PCR

RNA was isolated from sub-confluent growing cells with the use of High Pure RNA Isolation Kit (Roche, Germany; Cat. Nr. 11828665001) and 1 µg RNA was used for first-strand cDNA synthesis by using the RevertAid First Strand cDNA Synthesis Kit (Thermo Scientific, USA; Cat. Nr. K1622) according to the manufacturer's protocols. Serial two-fold dilutions of cDNA were prepared and amplified by PCR in order to ensure analysis in the exponential phase of the PCR reaction. Initial denaturation for all PCR reactions was 2.5 min at 95°C. Initial dilutions of cDNA for each reaction, primer sequences and conditions used for PCR amplifications are given in Table 1. The PCR products obtained by primers specific for *rps18* were used as loading controls. All PCR products were resolved by electrophoresis on 1.2% agarose gels, stained with ethidium bromide and visualized under ultraviolet light. The intensity of each band was determined by densitometry. For detection of Grp78 and CHOP mRNA expression Real-Time PCR was used. Real-Time PCR analysis was performed in triplicate on the QuantStudio 12K Flex Real-Time PCR System (Life Technologies) using the SYBR Green PCR Master mix (Life Technologies; Cat. Nr. 4309155). The PCR conditions were as follows: one cycle at 95°C for 10 min followed by 40 cycles at 95°C for 15 sec and at 60°C for 1 min. After amplification, dissociation curves were performed to ensure that a single PCR product had been amplified. For *Grp78*, the forward primer was 5′-GTTCTTGCCGTTCAAGGTGG-3′ and reverse was 5′-TGGTACAGTAACAACTGCATG-3′. For *CHOP*, the forward primer was 5′-ATGAGGACCTGCAAGAGGTCC-3′ and the reverse was 5′-TCCTCCTCAGTCAGCCAAGC-3′. For GAPDH, the forward primer was 5′-TGCACCACCACTGCTTAGC-3′ and the reverse was 5′-GGCATGGACTGTGGTCATGAG-3′. All the reactions were performed in triplicate, and the QuantStudio 12K Flex Software v1.0 was used for the quantification of the expression for each segment. Glyceraldehyde 3-phosphate dehydrogenase (*GAPDH*) was used as a normalization control gene.

**Table 1:** Primer sequences and conditions used for PCR amplification.

Gene	Sequence (5′ to 3′)	Denaturation	Annealing	Extension	Cycle	cDNA
					no.	dilution
ctr1	F taagattcggagagagaggtgc	30 s at 95°C	30 s at 63°C	30 s at 72°C	25	4-fold
	R aggctctctcggggctatctt					
nhe 1	F ccagtcattgccttctacc	30 s at 95°C	45 s at 63°C	60 s at 72°C	30	4-fold
	R tgtgtctgttgtaggaccgc					
atp7a	F cagttcaagacaaggaggaagg	30 s at 95°C	45 s at 63°C	60 s at 72°C	30	8-fold
	R tgtgctttgttggttgccagg					
mrp2	F gaacaattgtagagaaaggatc	40 s at 95°C	40 s at 52°C	90 s at 72°C	31	8-fold
	R cacaaacgcaaggatgatgaagaa					
rps18	F gtgtgctggcctcggacacg	45 s at 95°C	30 s at 61°C	30 s at 72°C	29	20-fold
	R caacatcgatgggcggcgga					

### Determination of reactive oxidative species

Generation of ROS was determined using the fluorescent dye 5-(and-6)-chloromethyl-2′,7′-dichlorodihydrofluorescein diacetate, acetyl ester (CM-H_2_DCFDA) (Life Technologies; Cat. Nr. C6827). Briefly, logarithmically growing cells were incubated with 10 µM CM-H_2_DCFDA for 1 h according to manufacturer's instructions. Afterward, the medium with dye was removed and cells were incubated in fresh medium with 40 µg/mL CBP for the indicated time periods. After trypsinization and centrifugation, cells were fixed in cold 80% methanol. Shortly before measurement, cells were centrifuged and resuspended in PBS. The fluorescence of the product, which is formed upon removal of the acetate groups from CM-H_2_DCFDA by ROS, was measured by flow cytometry. Incubation of cells with 0.1% H_2_O_2_ for 30 min was used as the positive control.

### Determination of DNA platination and total cell platination

To investigate the DNA platination level in parental HEp2 cells and HEp2-derived CBP-resistant 7T subline, cells were treated with different concentrations of CBP for various periods of time. The cells were harvested and DNA was isolated using QIAamp DNA Blood Mini (Qiagen, USA; Cat. Nr. 51104) according to the manufacturer's protocol. The concentration of isolated DNA was measured with Shimadzu-BiospecNano (Shimadzu Biotech, Germany) and the amount of platinum (Pt) atoms bound to DNA was measured by a validated high resolution inductively coupled plasma mass spectrometry (HR ICPMS) using the Element 2 (Thermo Finnigan, Bremen, Germany). The platinum nucleotide content was calculated using the relative molar masses of platinum and nucleotides obtained from DNA concentration [27]. Calibration standards were prepared from platinum standard solution 1 g/L in 2 M HCl (Fluka Riedel-de Haën, Germany; Cat. Nr. 80964). Total cell platination was measured as described previously [28]. Briefly, the cells were rinsed with ice-cold PBS, and harvested into 10 ml of ice-cold PBS using a rubber policeman. After centrifugation, the cells were resuspended in PBS, an aliquot was used for determination of cell number, and the remainder was digested in 70% nitric acid. Cell lysates were heated for 2 h at 75°C, diluted to 5% nitric acid, and assayed for platinum content.

### Gene silencing

For silencing of CHOP gene, the predesigned 100 nM of CHOP-specific siRNA (Silencer Select Predesigned siRNA; Ambion, USA; locus ID 1649) and control nonspecific siRNA for human (Silencer Select Predesigned siRNA Negative Control #1 siRNA; Ambion) were used. The transfection of siRNA was performed using Lipofectamine RNAiMAX Reagent (Life Technologies; Cat. Nr. 13778) according to the manufacturer's instructions in parental HEp2 and CBP-resistant 7T cells. 48 h after transfection the transfected cells were tested for CHOP expression by western blot and plated for assessment of cell survival by MTT assay or cell death by fluorescein diacetate and propidium iodide staining.

### Statistical analysis

All data were analyzed by unpaired Student's *t*-test and expressed as the mean ±standard error of the mean. Data were considered significant when *P*<0.05. All experiments were performed in triplicate and repeated at least two times.

## Results

### CBP-induced apoptosis is reduced in CBP-resistant 7T cells

**Figure 1:**
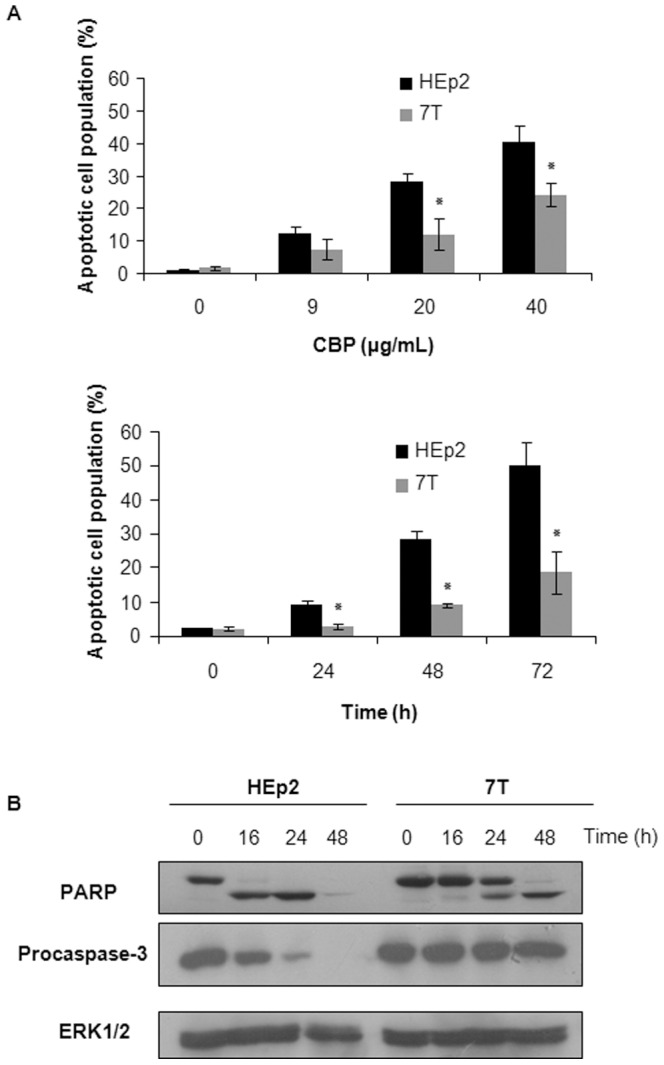
Induction of apoptosis in HEp2 and 7T cells following the CBP treatment. (A) The logarithmically growing cells were treated either with different doses of CBP during 72 h (upper panel) or with 40 µg/mL CBP for indicated time points (lower panel). Cells were stained with fluorescein diacetate and propidium iodide and examined by fluorescence microscopy. (B) Cleavage of PARP and pro-caspase 3 in HEp2 and 7T cell lines upon 48 h treatment with 40 µg/mL CBP. Representative data of three independent experiments are presented (mean ±SD). Control cells were collected 24 h after the beginning of the treatment. *p<0.05.

The 7T cell subline resistant to CBP was developed from HEp2 cells by stepwise exposure to CBP [22]. In order to determine the type of CBP-induced cell death, logarithmically growing HEp2 and 7T cells were treated continuously for 72 h with 9, 20 and 40 µg/mL of CBP or with 40 µg/mL CBP for 24, 48 and 72 h. In dose- and time-course experiments a reduced frequency of apoptotic cells following CBP treatment was observed in 7T cells as compared to parental HEp2 cell line (Fig. 1A). Furthermore, apoptotic cells in sensitive HEp2 cells were visible following the 24 h incubation, but not in 7T cells. The proportions of necrotic cells were similar in both cell types (data not shown). Thus, CBP resistance in 7T cells is associated with reduced induction of apoptosis. Protection of 7T cells from apoptosis following treatment with CBP was confirmed upon treatment of HEp2 and 7T cells with 40 µg/mL CBP for 24–48 h (Fig. 1A, lower figure).

Fig. 1B shows that cleavage of PARP in HEp2 cells was detectable 16 and 24 h after continuous exposure to CBP. Following a 48 h incubation, PARP protein was only weakly detectable in HEp2 cells, indicating that the majority of cells progressed to cell death. By contrast, cleavage of PARP in 7T cells occurred not before 24 h after the start of CBP treatment. Notably, PARP protein was still clearly detectable at the 48 h time point (Fig. 1B). These findings are in agreement with the results shown in Fig. 1A. Similar results were obtained upon western blot-based determination of procaspase-3 cleavage. The cleavage of procaspase-3 in HEp2 cells occurred 16 h after CBP treatment, and the procaspase-3 protein disappeared completely within 48 h, demonstrating that HEp2 cells underwent apoptosis. Cleavage of procaspase-3 in 7T cells was not detectable during the investigated time course (Fig. 1B). These results demonstrate that continuous exposure to CBP induces apoptosis in both HEp2 and 7T cells. Yet, cell death is triggered earlier and with higher efficacy in the parental HEp2 cells.

### Resistant 7T cells reveal reduced CBP accumulation

**Figure 2:**
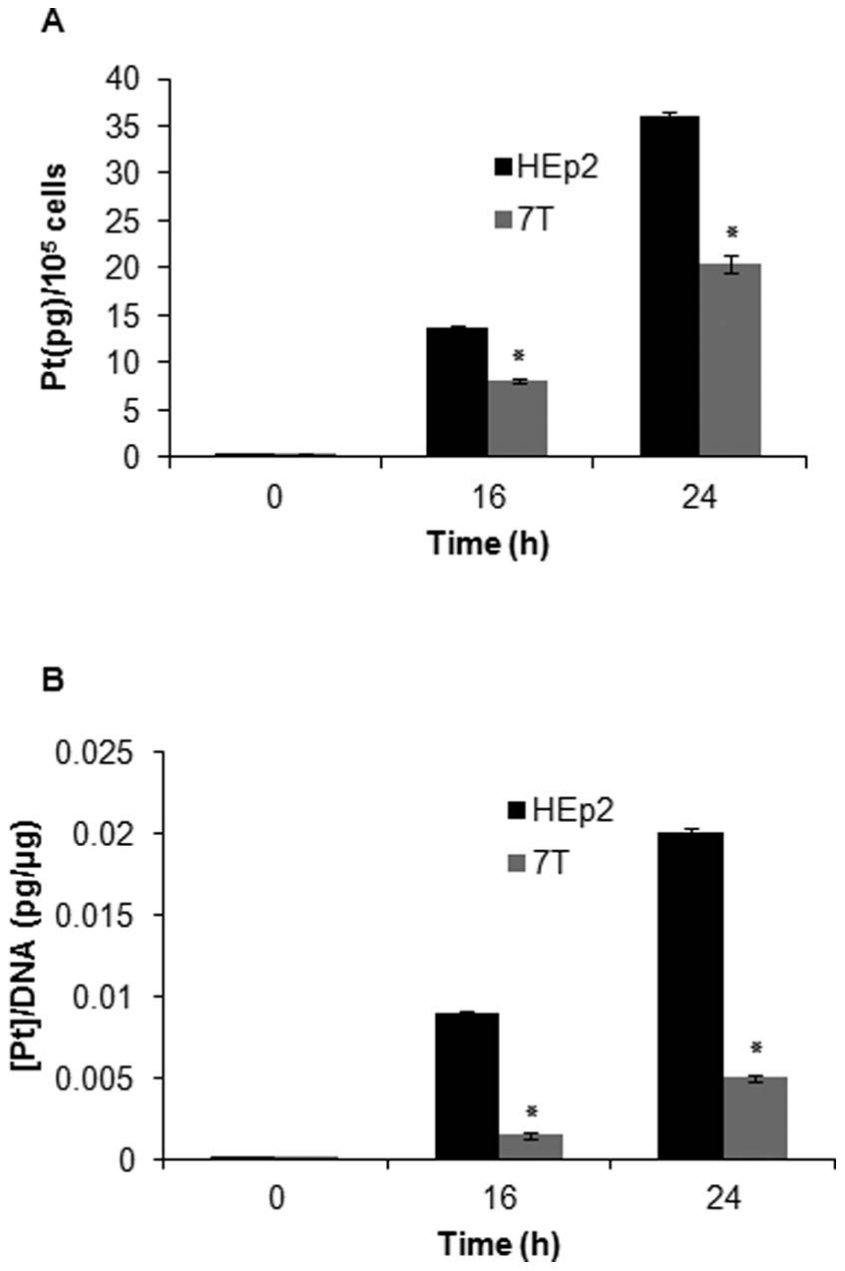
Total and DNA-platination in HEp2 and 7T cells. (A) The logarithmically grown cells were treated with 40 µg/mL CBP. The adherent cell population was collected at the indicated time points and total cell platination was measured. (B) The logarithmically grown cells were treated with 40 µg/mL CBP and the adherent cell population was collected, DNA was isolated, and DNA-platination was measured. Representative data of three independent experiments are presented (mean ±SD). *p<0.05.

In Pt-drug resistant cells a reduced intracellular accumulation of the drug has often been observed [5], [6], [7]. To determine whether the increased survival of 7T cells upon CBP treatment was linked to changes in intracellular Pt content, total cell platination was measured by ICPMS, following overnight exposure with 40 µg/mL CBP. The data, normalized to Pt standard, showed a reduced Pt accumulation in 7T as compared to HEp2 cells (Fig. 2A) and a lower level of DNA-Pt adducts (∼about 6 fold less in 7T *versus* HEp2 cells) (Fig. 2B). Therefore, our results show that 7T cells accumulate less Pt, resulting in a lower level of DNA-Pt adducts as compared to parental HEp2 cells.

### CBP induces less DNA lesions in resistant 7T cells

**Figure 3:**
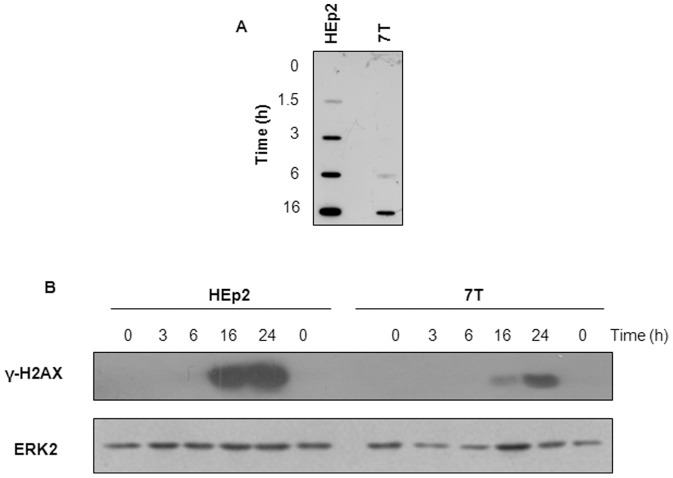
DNA lesions induced by CBP in HEp2 and 7T cells. (A) Upon treatment with 40 µg/mL CBP the cells were collected at the indicated time points and DNA was isolated and transferred on nitrocellulose membrane. After fixation the membrane was incubated with antibody specifically recognizing 1,2-GG intrastrand cross-links induced by CBP. (B) The logarithmically growing cells were treated during indicated time points with 40 µg/mL CBP. The phosphorylation of H2AX (γH2AX) was investigated by Western blot analysis. Expression of ERK2 was used as an internal loading control. Representative data of two independent experiments are presented. Control cells were collected 24 h after the beginning of the treatment.

In order to determine whether the difference in intracellular accumulation of platinum observed in HEp2 and 7T cells was detectable at the level of DNA as well, we measured the kinetics of formation of DNA intrastrand and DNA interstrand cross-links induced by CBP in HEp2 cells and its CBP-resistant 7T subline. DNA-Pt adducts were detectable 1.5 h after CBP treatment in HEp2 cells, whereas a similar content of DNA-Pt adducts in the 7T cell line was attained after a 6 h incubation (Fig. 3A). The different levels of intrastrand DNA-Pt adducts in parental HEp2 and CBP-resistant 7T cells were detected up to 16 h after CBP treatment (Fig. 3A). Densitometric analysis at 16 h reveals about 2.3-fold less DNA-Pt adducts in 7T as compared to HEp2 cells. In order to assess indirectly the formation of interstrand cross-links we measured the level of phospho-H2AX (γH2AX) expression accepted as a surrogate marker of DNA double-strand breaks formed during the processing of interstrand cross-links [29]. CBP-induced double-strand breaks were measured in cells treated for 3–24 h with 40 µg/mL CBP. Increased expression of γH2AX was detectable 16 and 24 h after CBP treatment in both, HEp2 and 7T cells, but the level of γH2AX was much higher in parental HEp2 than in CBP-resistant 7T cells. Densitometric analysis at 16 h showed about 4.4-fold lower expression of γH2AX in 7T as compared to HEp2 cells (Fig. 3B). Identical results were obtained 48 h post drug treatment (data not shown). The reduced level of γH2AX in 7T cells is probably the result of a lower amount of interstrand cross-links. These results are in the line with the lower level of DNA platination observed in 7T cells (see Fig. 2B).

During treatment with CBP, the DNA-Pt adducts can be repaired by different types of DNA repair systems such as nucleotide excision repair (NER), base excision repair (BER), mismatch repair (MMR), and double-strand break repair (DSBR) [5], [30]. Therefore, adaptive changes in the expression of proteins involved in DNA repair could be involved in acquired resistance to carboplatin [5], [11]. Yet, we detected no differences between parental HEp2 and resistant 7T cells in the constitutive expression of Ercc1 and XPF (proteins involved in NER), as well as in the expression of MutL and MutS (proteins involved in MMR), which are of relevance for platinum sensitivity [6] (data not shown). Therefore, the decreased levels of platinum-DNA cross-links are not likely due to changes in DNA repair, but rather decreased accumulation of platinum in CBP-resistant cells.

To summarize, decreased whole cell platination was observed in the CBP-resistant 7T relative to parental HEp2 cells, and this is associated with a lower amount of DNA intra- and interstrand cross-links.

### Expression of CTR1, NHE1, ATP7A and MRP2 mRNAs is changed in CBP-resistant cells

**Figure 4:**
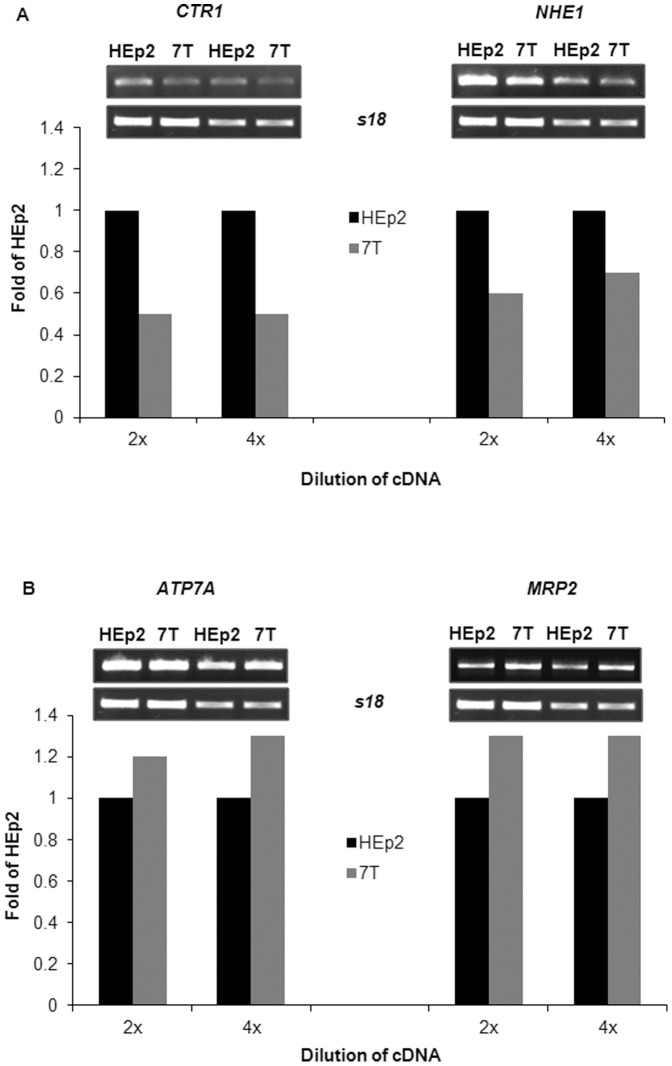
mRNA expression of *CTR1*, *NHE1*, *ATP7A* and *MRP2* in HEp2 and 7T cells. The logarithmically growing cells were collected and RNA was isolated. Semi-quantitative RT-PCR and densitometry analysis of bands were performed. As an internal control for equal RNA/cDNA loading, *s18* was used. The representative of 3 independently isolated RNAs and independently performed RT-PCRs are presented. (A) influx pumps *CTR1*and *NHE1*, (B) efflux pumps *ATP7A* and *MRP2*.

Decreases in total cell and DNA platination in 7T cells might be the result of an altered expression of drug transporter proteins/pumps located in the cell's or organelles' membranes [5], [6], [7], [31]. Therefore, mRNA expression of platin influx pumps, namely the copper transporter (*CTR1*) and the Na(+)/H(+) exchanger isoform 1 (*NHE1*), as well as of efflux pumps, such as the copper-transporting P-type adenosine triphosphatase (*ATP7*) and the multidrug resistance associated proteins 1 (*MRP2*), were measured. Fig. 4A shows that the mRNA expression of the influx pumps *CTR1* and *NHE1* was reduced by 50% and 30%, respectively, in CBP-resistant 7T as compared to parental HEp2 cells. The expression of the efflux pumps *ATP7A* and *MRP2* was slightly increased in 7T as compared to parental HEp2 cells (Fig. 4B). Thus, the reduced DNA platination of CBP-resistant 7T cells very likely results from altered expression of transporter proteins.

### CBP treatment generates less ROS in resistant 7T cells

**Figure 5:**
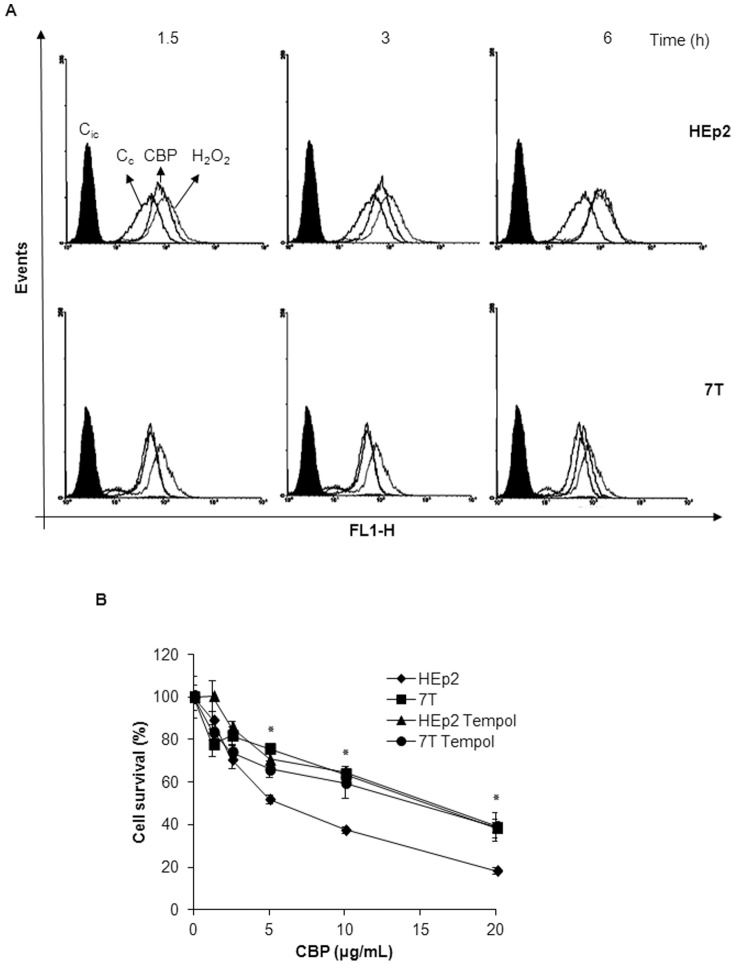
CBP induced ROS formation in HEp2 and 7T cells. (A) The logarithmically growing cell lines were treated with 40 µg/mL CBP during the indicated period of time. The cells were collected, stained with CM-H_2_DCFDA and ROS was measured by flow cytometry. (B) The HEp2 and 7T cells were pretreated with 0.1 mM tempol. Two hours later different concentrations of CBP were added. The cell survival was determined 72 hours later by MTT assay. Representative data of three independent experiments are presented (mean ±SD). *p<0.05. C_ic_-isotype control, C_c_-untreated cells, CBP-carboplatin treated cells, H_2_O_2_-hydrogen peroxide treated cells.

Generation of ROS has been demonstrated to be an early event that triggers apoptosis [32], [33]. As shown in Fig. 5A, exposure of HEp2 and 7T cells to 40 µg/mL CBP resulted in a time-dependent increase of ROS formation compared with DMSO-treated controls. ROS formation was significantly increased as early as 1.5 h after treatment with CBP in parental HEp2 cells, while the CBP-mediated increase in ROS formation in CBP-resistant 7T cells was first detected 6 h after CBP treatment. These results suggest that CBP causes ROS formation very early after treatment in HEp2 cells and that the generation of ROS is diminished in the HEp2-derived CBP-resistant 7T cell subline, probably due to decreased accumulation of platinum in 7T cells.

In order to assess the role of ROS formation in the response of HEp2 and 7T cells to CBP we used ROS scavenger tempol. Pretreatment of cells with 0.1 mM of tempol, followed by CBP treatment resulted in an increased cell survival of HEp2 cells, showing the importance of ROS generation in CBP-induced cell death. However, tempol did not have such an effect in 7T cells which implies that, although triggered, ROS formation is not involved in response of 7T cells to CBP (Fig. 5B). A similar result was obtained with another ROS scavenger, N-acetylcysteine (data not shown).

### CBP induced less ER stress in CBP-resistant cells

**Figure 6:**
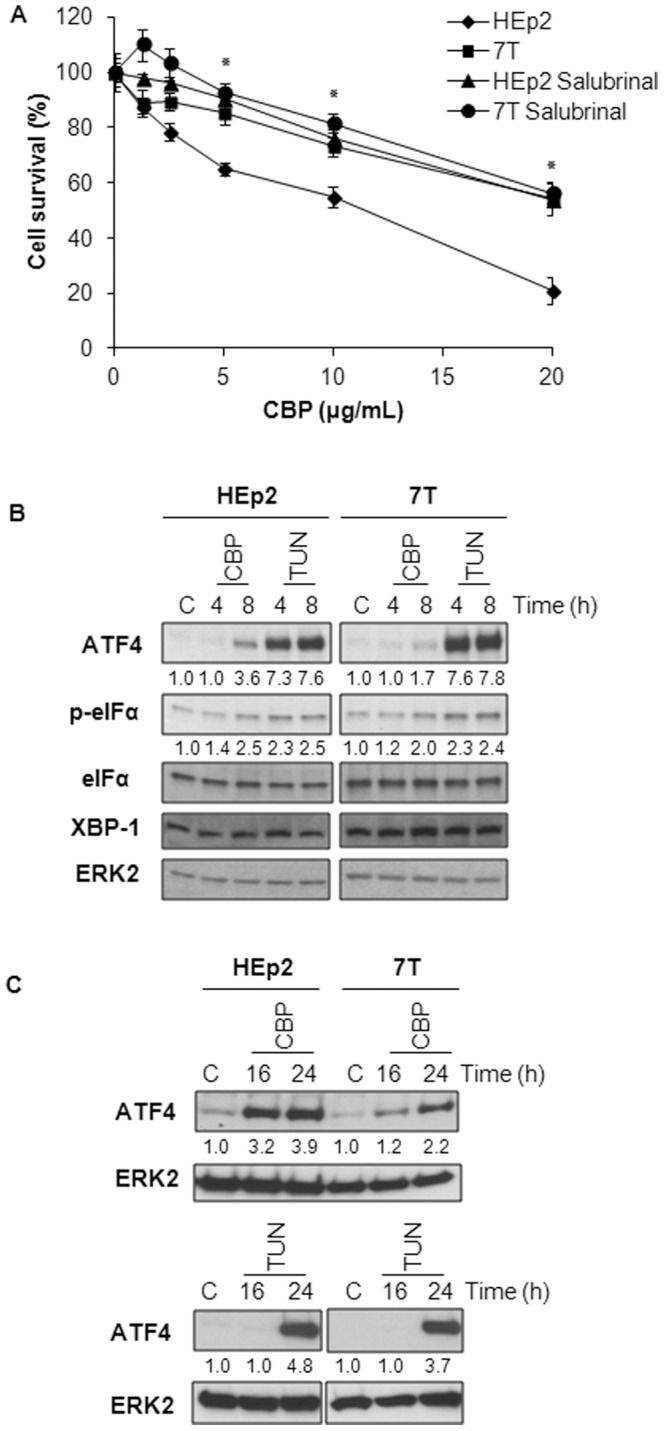
CBP induced ER stress in HEp2 and 7T cells. (A) HEp2 and 7T cells were pretreated for two hours with 12.5 µM salubrinal, and then treated with different concentrations of CBP. Seventy-two hours later cell survival was measured by MTT assay. Representative data of three independent experiments are presented (mean ±SD). *Significantly different from the respective control. * p<0.05. (B) HEp2 and 7T cells were treated with 80 µg/mL CBP or 10 µg/mL tunicamycin for 4 and 8 h. At indicated time points the cells were collected and the expression of ATF4, phospho-eIFα (p-eIFα), eIFα and XBP-1 was determined. (C) HEp2 and 7T cells were treated with 40 µg/mL CBP or 1 µg/mL tunicamycin during 16 and 24 h. At indicated time points the cells were collected and the expression of ATF4 was determined. ERK2 control was used as an internal loading control. Data of one of two experiments that yielded similar results are presented. Densitometric analysis data are expressed as a ratio between examined ER markers and ERK2. Non treated cells were set as 1.0.

High amounts of ROS can cause damage to proteins leading to the so-called unfolded protein/ER stress response [34]. Since ROS was detected very early upon CBP treatment in HEp2 cells (observed as early as 1.5 h after CBP treatment), we hypothesize that CBP-induced ROS could trigger ER stress. To investigate the possible role of ER stress in response to CBP treatment of HEp2 and 7T cells we pretreated both cell lines with a specific inhibitor of ER stress, salubrinal. In HEp2 cells salubrinal increased the survival following the treatment with CBP to the levels of the CBP-resistant 7T subline (Fig. 6A) indicating the involvement of ER stress in CBP response, as well as its role in mediating sensitivity to CBP. However, pretreatment with salubrinal did not have any effect in 7T cells (Fig. 6A) indicating that in these cells ER stress is not involved in a CBP response.

To clarify further the role of CBP-triggered ER stress in our model system we treated cells with either high doses of CBP (80 µg/mL) or tunicamycin (10 µg/mL) for a short period of time (4 and 8 hours) and measured the levels of several typical inducible ER stress marker proteins: ATF4, p-eIFα and XBP-1 [35]. The idea of using the well-known ER stress inducer tunicamycin is to assess the ability of induction of ER stress in HEp2 and 7T cells by the measurement of ER stress marker proteins. As presented in Fig. 6B, the treatment with 80 µg/mL CBP during 4 and 8 h resulted in increased expression of ATF4 and p-eIFα in both HEp2 and 7T cells. However, the induction of these ER stress markers, especially ATF4, was higher in HEp2 than in 7T cells. The differences in intensity of ATF4 and p-eIFα expression between HEp2 and 7T cells are probably a result of different Pt level in the cells. The potential of ER stress induction was comparable in both cell lines since 10 µg/mL tunicamycin treatment for 4 and 8 h similarly upregulated the expression of ATF4 and p-eIFα. In neither of the cell lines upon CBP or tunicamycin treatment we detected any differences in the expression of eIFα and XBP-1 as compared to untreated controls.

Since the most obvious increase of protein expression upon CBP and tunicamycin treatment was detected at the level of ATF4 protein we decided to analyze differential response of HEp2 and 7T cells to CBP along with tunicamycin as a control treatment, at a later time, i.e. 16 and 24 hours and upon lower doses of CBP (40 µg/mL) and tunicamycin (1 µg/mL). Data in Fig. 6C confirmed the data from Fig. 6B showing that CBP induces ATF4 more noticeably and somewhat earlier in HEp2 compared to 7T cells. Concomitantly, ATF4 induction by 1 µg/mL tunicamycin was slightly higher in HEp2 than in 7T cells (Fig. 6C).

To summarize, although in HEp2 and 7T cell lines the ER stress can be successfully induced, as shown by the treatment with tunicamycin, we observed less pronounced upregulation of the ER stress markers upon the CBP treatment in 7T than in HEp2 cells, probably as a consequence of the lower intracellular content of CBP and lesser ROS generation in 7T as compared to HEp2 cells. However, while salubrinal has a strong effect on HEp2 survival upon CBP treatment, an effect which is absent in CBP-resistant cell line 7T, we can conclude that ER stress is involved in the response to CBP in parental HEp2 cells but not in CBP-resistant 7T cells.

### CBP induces Grp78 and CHOP expression at the mRNA and protein levels

**Figure 7:**
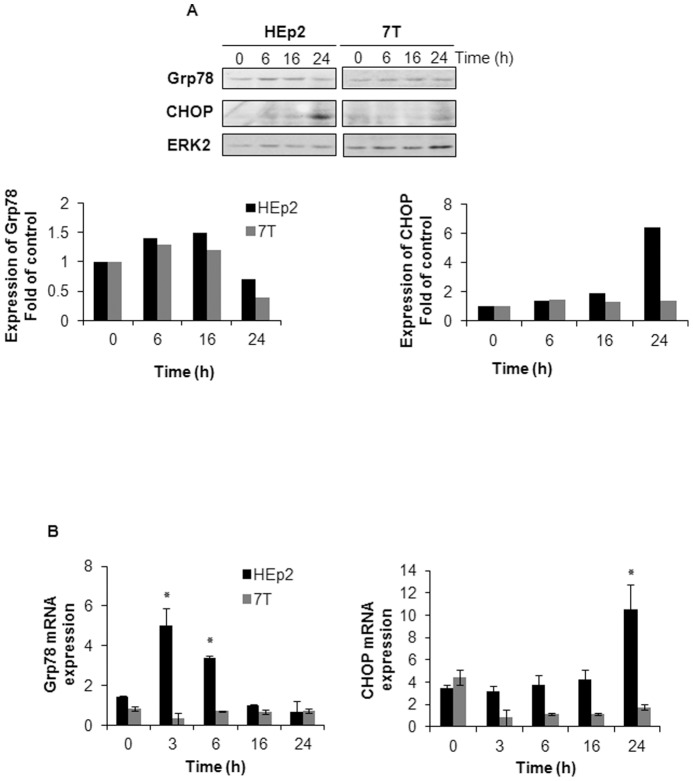
CBP induced Grp78 and CHOP expression in HEp2 but not in 7T cells. (A) Twenty-four hours after the seeding, HEp2 and 7T cells were treated with 40 µg/mL CBP. At indicated time points the cells were collected and the expression of ER stress markers CHOP and Grp78 was determined. Expression of ERK2 was used as an internal loading control. (B) HEp2 and 7T cells were treated with 40 µg/mL CBP for 3–24 h. At indicated time points the cells were collected and the mRNA expression of ER stress markers CHOP and Grp78 was determined. Expression of GAPDH was used as an internal control.

We next analyzed the downstream key proteins of ER stress response, the ER stress markers CHOP and Grp78. It is known that in the case of prolonged or severe ER stress, the apoptotic process is triggered to eliminate damaged cells [36] and that Grp78 and CHOP expression increase during that event [37]. 24 h after CBP treatment HEp2 cells showed an increase in CHOP expression as compared to 7T cells. We detected slightly higher levels of Grp78 protein 16 and 24 h after CBP treatment in HEp2 as compared to 7T cells (Fig. 7A). The increased expression of CHOP and Grp78 proteins was confirmed at the mRNA level upon CBP treatment. Our results show that CBP-resistant 7T cells display similar mRNA expression of Grp78 and CHOP detected during different times of drug exposure. However, very early upon CBP exposure (3 and 6 h), Grp78 mRNA expression increased up to 2–3-fold compared to untreated cells in the parental HEp2 cell line and then dropped to a constitutive level in later time points. CHOP mRNA expression increased 3-fold compared to untreated cells 24 h upon drug treatment in HEp2 cells (Fig. 7B).

### CHOP is involved in response of HEp2 cells to carboplatin

**Figure 8:**
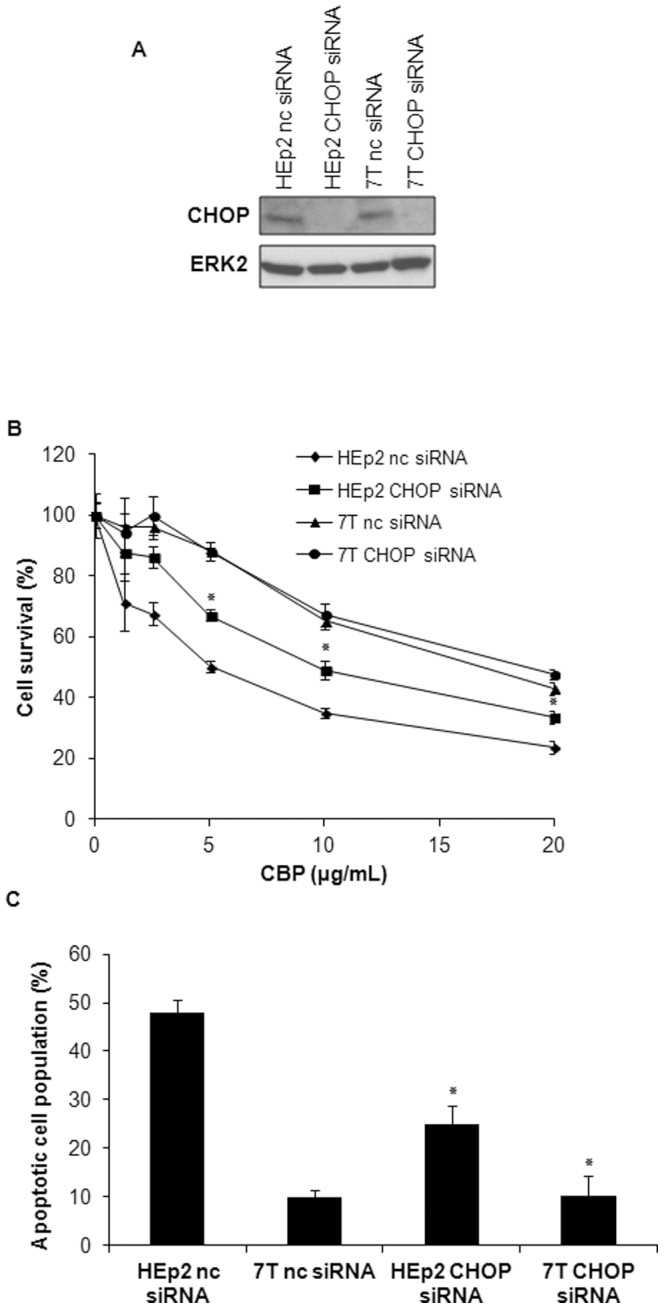
CHOP is involved in response of HEp2 cells to CBP. (A) Parental HEp2 and CBP-resistant 7T cells were transfected with negative control siRNA (nc siRNA) or with CHOP-specific siRNA (CHOP siRNA) The expression of CHOP was determined by western blot 48 h after transfection. ERK2 was used as equal loading control. Representative blot of two independent experiments that yielded similar results is presented. (B) HEp2 and 7T cells were seeded for MTT assay 48 h after transfection with nc siRNA or CHOP siRNA and treated with CBP 24 h after. MTT assay data are representative of two independently performed experiments ±S.D. (C) HEp2 and 7T cells were transfected with nc siRNA or CHOP siRNA, plated and 24 h later treated with 40 µg/mL CBP for 48 h. Cells were stained with fluorescein diacetate and propidium iodide and examined by fluorescence microscopy. Representative data of three independent experiments are presented (mean ±SD). *p<0.05.

Since the protein expression of CHOP was more evident than Grp78 we decided to silence CHOP in order to examine its role in sensitivity of HEp2 and 7T cells to CBP. The silencing of CHOP gene upon transfection of HEp2 and 7T cells with CHOP-specific siRNA was successful (Fig. 8A). Transfection of HEp2 and 7T cells with negative control (nc) siRNA did not influence sensitivity to CBP as compared to non transfected cells (data not shown). Upon establishment of successful CHOP silencing, the sensitivity of HEp2 and 7T cells upon CBP treatment was determined using the MTT assay. Silencing of CHOP in 7T cells did not change survival of 7T cells. However, silencing of CHOP in HEp2 cells significantly increased survival, i.e. induced resistance to CBP as compared to cells transfected with control nc siRNA (Fig. 8B). These results were confirmed with measurement of the percentage of apoptotic cell population specifically 48 h upon CBP treatment (Fig. 8C). The silencing of CHOP in HEp2 cells significantly decreased the percentage of apoptotic cell population upon CBP treatment, while this effect was absent in CBP-resistant cells 7T. Overall, obtained results show that CHOP plays a significant role in response of HEp2 to CBP while such an effect does not exist in 7T cells.

## Discussion

Acquired resistance is a major obstacle to successful chemotherapy. It is based on multiple molecular alterations that often cause resistance not only to the drug to which the cells were exposed, but also to unrelated anti-cancer drugs. In this work we use as a model 7T cells obtained by multiple exposures of HEp2 cells to CBP that were cross-resistant to cisplatin, transplatin and mitomycine C, while their sensitivity to doxorubicin, etoposide, camptothecin, vincristine, paclitaxel and methylmethanesulfonate did not change as compared to parental cells. Here we show that CBP-resistant 7T cells display reduced CBP-induced apoptosis as compared to parental HEp2 cells. Necrotic cell death was not affected (data not shown). Similar results have been shown in other cell models, indicating that changes in apoptosis induction significantly contribute to a CBP-resistant phenotype [38], [39].

The intracellular Pt-level, notably DNA platination, can determine the final outcome of the treatment with platinum drugs as described previously (for instance [24]). Therefore, to explain the lower frequency of apoptotic cells upon the CBP treatment in 7T cells, we measured both the total cell and DNA platination in HEp2 and 7T cells. The level of platinum detected in CBP-resistant 7T cells was lower at the level of both total cell and DNA platination compared to parental HEp2 cell line. In accordance, reduced level of specific 1,2-GG intrastrand cross-links was found upon CBP treatment in 7T cells. At a later time point, interstrand cross-links can appear, leading further to the formation of DNA double-strand breaks [40], [41]. In addition to its role in DNA-damage repair, H2AX is required for DNA fragmentation during apoptosis and is phosphorylated by various kinases in response to apoptotic signals [41]. Our results showed that the lower level of initial DNA platination in 7T cells resulted in a lower level of intrastrand cross-links and later in a lower level of DNA double-strand breaks as indicated by a decreased level of γH2AX protein expression. Previously, we obtained similar results regarding the induction and repair of platinum-DNA lesions in human cervical carcinoma cells resistant to cisplatin [24].

Less Pt detected in the CBP-resistant cells could be based on decreased influx and/or increased efflux of the drug. Alterations in the expression of different transporter proteins, such as *CTR1*, *ATP7A*, *ATP7B*, *NHE1*, and *MRP2* as well as changes in cell membrane structure can influence active and passive transport of the drug [42]. We detected slightly reduced mRNA level for *CTR1* and *NHE1* transporter influx pumps and increased expression of efflux pumps, *ATP7A* and *MRP2* in CBP-resistant 7T cell subline as compared to HEp2 cell line. Despite the fact that we did not measure the protein expression of different transporter proteins, our data suggest that altered transport of CBP, especially an increased efflux of Pt out of the cells is probably the main mechanism of resistance to CBP in 7T cells. The relevance of changes in drug transport for CBP resistance was demonstrated in murine embryonic fibroblast. Upon deletion of the *CTR1* gene (ctr1−/− MEFs), a 2-fold CBP resistance was detected [43]. Over-expression of *ATP7A* in ovarian carcinoma 2008 cells resulted in higher levels of Pt drug accumulation upon treatment with the platinum drugs cDDP, CBP and oxaliplatin. However, these *ATP7A* over-expressing cells are resistant to all three examined drugs, indicating that the function of *ATP7A* in cell response to platinum drugs is not reduction of accumulation but more likely the binding and sequestration of Pt drugs [44]. Finally, for the last examined transporter, *MRP2* was found to be overexpressed in human ovarian carcinoma A2780 cells resistant to CBP [45]. Yet we cannot exclude a possible role of other transporter proteins in CBP resistance, such as *ATP7B* [46]. Furthermore, the passive diffusion of Pt drugs through the cell membrane can be also altered in CBP-resistant cell lines as compared to parental cells [5]. Our results indicate that several platinum transporters are differentially expressed in CBP-resistant 7T cells due to exposure to CBP, resulting in lower intracellular CBP accumulation and decreased cell death relative to parental HEp2 cells.

Recent data has shown that cisplatin can induce ROS formation. In our recent review we presented and discussed the current knowledge on the molecular mechanism of cDDP-induced ROS formation, the relationship between ROS formation, the protective roles of GSH and BCL-2 protein, and highlight possible mechanisms that may lead to cDDP resistance [47]. Moreover, we demonstrated that ROS formation is triggered early following exposure to cDDP, and that cDDP resistance is associated with GSH-dependent elimination of drug-induced generation of ROS in integrin α_V_β_3_-overexpressing HEp2 cells [48]. In accordance, CBP-induced ROS formation is thought to be responsible for the oxidative injury in the cochleae of rats [14]. In addition, it is reported that CBP-induced cardiotoxicity is related to ROS production in mice and could be prevented by using pravastatin, which protected against oxidative stress [32]. Production of ROS is involved in CBP-induced damage to murine cochlear hair cells and spiral ganglion neurons, and can be attenuated by the antioxidant NAC [49].

In the present study we show the appearance of ROS at 1.5 h following CBP treatment in HEp2 cells, while it took 6 h before it was formed in 7T cells. The role of ROS formation in HEp2 and 7T cell response to CBP was examined by pretreatment of cells with an effective antioxidant tempol. Tempol increased survival of HEp2 upon CBP treatment as compared to HEp2 cells treated only with CBP implicating that generation of ROS impacts cell survival in cell response to CBP. Pretreatment with tempol did not change survival rate of 7T cells upon CBP treatment at all. It is important to note that CBP actually induces ROS in CBP-resistant cells 7T but a little bit later in time than in HEp2 cells, very likely due to the lower intracellular content of CBP. Therefore the inability of tempol to modulate cell survival in 7T cells indicate that formation of ROS is not involved in 7T cells response to CBP.

Different cytotoxic drugs can induce the generation of ROS which further triggers the ER stress response [50]. Namely, it was shown that cisplatin can induce nuclear damage-independent apoptosis in enucleated mouse kidney proximal tubule TKPTS cells [20], and in cytoplasts prepared from human melanoma cell line 224 and colon cancer HCT116 cell lines [19]. These data suggest that DNA damage-independent cDDP-induced signaling pathways are stimulated by oxidative stress. In this report we showed for the first time that CBP is able to induce ER stress. Our results show that ER stress signaling is triggered immediately as CBP enters the cell (4–8 h) since in both cell lines, HEp2 and 7T, we observed an increase in ATF4 and p-eIFα expression after drug treatment. However, the induction of these ER stress markers, especially ATF4, was higher in HEp2 than in 7T cells. As a control for the general ability of ER stress induction in HEp2 and 7T cells we used tunicamycin. Tunicamycin was able to evidently induce similar levels of ATF4 and in lesser extent, p-eIFα expression in HEp2 and 7T cell lines. Finally, the importance of ER stress induction upon CBP was assessed by pretreatment of HEp2 and 7T cells with salubrinal, a specific inhibitor of ER stress [51]. Namely, salubrinal increased survival rate upon CBP treatment in parental HEp2 cell line only, emphasizing again the involvement of ER-stress in cell response to CBP in HEp2 but not in CBP-resistant 7T cells.

The CBP treatment had differential effect on expression of downstream ER stress markers Grp78 and CHOP in HEp2 and 7T cells. While in HEp2 Grp78 mRNA increased 3 hours upon CBP treatment, it took 24 hours for CHOP mRNA upregulation. In CBP-resistant 7T cells no changes in Grp78 and CHOP mRNA levels were observed. However, the involvement of endoplasmatic reticulum stress in the response of HEp2 and 7T cells to CBP was further analyzed by specific silencing of CHOP gene in both cell lines. Namely, specific silencing of CHOP gene in HEp2 cells obtained by transfection with CHOP-specific siRNA increased survival of HEp2 cell line as compared to the HEp2 cells transfected with control nc siRNA. Similarly as observed upon tempol and salubrinal pretreatment, CHOP silencing did not have any effect on 7T cell response to CBP. Therefore, our data in HEp2 cells confirmed the role of ER stress in CBP toxicity. It seems that shortly upon entering the cell, CBP simultaneously causes DNA damages, triggers production of ROS, and activates ER stress. We showed that despite the well-accepted fact that DNA damages are important for CBP cell toxicity, ROS production plays an important role in this toxicity as well. Moreover, inhibiting the formation of ROS and ER stress pathway significantly altered the sensitivity of HEp2 cells to CBP, rendering them as resistant as 7T cells. Therefore we conclude that in HEp2 cells the ROS formation and ER stress induction both play an important role in sensitivity to CBP, and generally could have a role in CBP resistance. Nevertheless, inhibiting the formation of ROS and ER stress pathway did not change sensitivity of CBP-resistant 7T cells to CBP indicating that in these cells CBP toxicity became independent on ROS induction and of ER stress.

In summary, our results suggest that in HEp2 cells CBP-induced ROS is a stimulus for ER stress, which together with DNA lesions, triggers cell death by apoptosis. Contrary, despite the ability of CBP to induce formation of ROS and activate ER stress in 7T cells, the cell death mechanism in 7T cells is independent of ROS induction and activation of ER stress. Our results contribute to the knowledge about the variety of mechanisms of cell death to antitumor drugs, especially for drugs such as CBP, which can act at multiple levels in a cell. In addition, the novel signaling pathway of CBP-driven toxicity in the HEp2 cell line, i.e. ROS formation and induction of ER stress may be a potential target for future modulation of CBP-based therapy of epithelial cancers and it will be interesting to determinate whether a similar mechanism exists *in vivo*.
